# The Reliability of Salivary Cortisol Compared to Serum Cortisol for Diagnosing Adrenal Insufficiency with the Gold Standard ACTH Stimulation Test in Children

**DOI:** 10.3390/children10091569

**Published:** 2023-09-19

**Authors:** Silvia Ciancia, Sjoerd A. A. van den Berg, Erica L. T. van den Akker

**Affiliations:** 1 Division of Endocrinology, Department of Pediatrics, Erasmus MC-Sophia Children’s Hospital, University Medical Center, 3015 Rotterdam, The Netherlands; 2 Department of Clinical Chemistry, Erasmus University Medical Center, 3015 Rotterdam, The Netherlands

**Keywords:** high-dose ACTH stimulation test, low-dose ACTH stimulation test, salivary cortisol, children

## Abstract

The ACTH (adrenocorticotropic hormone) stimulation test is the gold standard for the diagnosis of adrenal insufficiency (AI), performed with ACTH high dose (HDT) or low dose (LDT). As salivary cortisol has been proposed as an alternative to serum cortisol, our aim was to evaluate the reliability of salivary cortisol compared to serum cortisol for diagnosing AI in children. Data were collected retrospectively. Salivary and serum cortisol values derived by 80 ACTH stimulation tests were obtained (39 F, 36 M; median age 11.5 years, IQR 6.9). Sampling was performed at baseline and after 30 and 60 min from ACTH administration during the HDT, and at baseline and 10, 20, 30, 40 and 60 min after the stimulation for the LDT. A serum cortisol level > 420 nmol/L ruled out AI. The correlation coefficients between serum and salivary cortisol for the HDT (*n* = 24) were 0.80 at t0, 0.48 at t30 and 0.75 at t60. All patients were adrenal sufficient. In 41% of the LDT, peak serum cortisol indicated insufficient adrenal function. The correlation coefficients between serum and salivary cortisol were 0.59 at t0 and 0.33 at the peak. For a cut-off of salivary cortisol < 15 nmol/L, sensitivity was 73.9% and specificity 69.6%. Our data do not support salivary cortisol as a valid alternative to serum cortisol during LDT. Regarding the HDT, results are more encouraging, however, further studies are needed.

## 1. Introduction

Adrenal insufficiency (AI) is determined by the dysfunction of the cortex of adrenal glands, where cortisol is synthetized [1]. AI can be classified as primary (PAI), due to dysfunction/disruption of the adrenal glands; secondary (SAI), caused by ACTH deficiency as part of multiple pituitary hormone deficiency or in an isolated form, or tertiary, caused by impaired hypothalamic secretion of CRH, usually due to long-term treatment with exogenous steroids [1]. Epidemiological data about the incidence of AI are not uniform according to different studies and have been estimated in adults [1,2,3]. In children, AI is more rare and only the incidence of congenital adrenal hyperplasia (CAH) is available (1/10,000 to 1/18,000), being the most common cause of PAI in this population [2]. In 11.3% of pediatric cases, PAI is caused by rare congenital diseases [4].

AI has a high impact on the life of patients and increases the risk of mortality after adrenal crisis. A study conducted in Israel on 120 children affected by AI reported an incidence of adrenal crisis of 3.4/100 patients/year, increasing to 6.6/100 patients/year in patients younger than 7 years [5]. Therefore, a correct and timely diagnosis of AI is essential.

A random measurement of basal cortisol levels does not reflect cortisol production reliably because cortisol secretion is characterized by a circadian rhythm, and serum cortisol levels oscillate during the 24 h [6]. The gold standard for the diagnosis of PAI is the ACTH stimulation test, performed with the administration of the standard dose of i.v. Synachten^®^ 250 µg for adults and children aged ≥2 years. AI is diagnosed if the peak cortisol level is below 500 nmol/L (18 µg/dL) [7]; however, this cut-off is assay dependent, thus every laboratory should provide a specific threshold value [8]. A Synachten^®^ dose of 1 µg is used as an alternative to the standard dose ACTH stimulation test in many clinical settings because several studies have shown that both high-dose ACTH test (HDT) and low-dose ACTH test (LDT) have a similar diagnostic accuracy [9].

In the last years, salivary cortisol has been proposed as an alternative to serum cortisol. Cortisol spreads from blood to saliva passively, responding to changes in plasma cortisol concentration quickly. Salivary cortisol concentration is independent from salivary flux and it reflects free serum cortisol, therefore the determination of salivary cortisol levels can be helpful, for example, in patients with low levels of cortisol binding globulin (CGB), in whom total serum cortisol could be “falsely” low, or in patients with high levels of CBG (such as patients assuming oral contraceptives), in which serum cortisol could be “falsely” normal [8]. The collection of a salivary sample is easy, non-invasive and can be conducted passively (drooling into a container) or actively (chewing a cotton pad). In both cases, patients should not eat, drink, smoke or brush teeth for at least the previous 30 min. Salivary cortisol concentration is less than one-tenth of that in serum, thus blood contamination of the salivary sample must be avoided [10,11].

A good correlation between serum and salivary cortisol has been shown and the value of 13.2 nmol/L has been proposed as the threshold to discriminate among adrenal insufficient (AI) and adrenal sufficient (AS) patients [12] [13]. A different cut-off has been suggested by Langelaan et al., according to whom an early morning salivary cortisol >5.9 nmol/L can rule out AI; in patients presenting lower values, an HDT is recommended (cut-off of peak salivary cortisol 17.2 nmol/L) [14]. However, the data were obtained from an adult population and salivary cortisol was determined by liquid chromatography tandem mass spectrometry (LC-MS/MS) rather than immunoassay. Chao et al. tested 21 children aged 6–17 years with 250 µg of ACTH administered intravenously or intramuscularly. Serum and salivary samples were collected at baseline and after 45 min. Based on their analysis, stimulated salivary cortisol below 200 ng/dl (5,5 nmol/L or 10 µg/dL) would diagnose AI, a value above 500 ng/dl (13,8 nmol/L or 18 µg/dL) would rule out AI and values between 200 and 500 ng/dl would indicate partial AI [15].

Clearly, protocols for the ACTH stimulation test are not homogenous and different salivary cortisol cut-offs have been derived. In addition, data in children are very limited.

In the current study, the aim was to evaluate the reliability of salivary cortisol compared to serum cortisol for diagnosing AI in children who underwent the ACTH stimulation test, both HDT and LDT. The correlation between serum and salivary cortisol was evaluated at baseline and at the peak, and the sensitivity, specificity, positive predictive value (PPV) and negative predictive value (NPV) of salivary cortisol levels for the diagnosis of AI were calculated.

## 2. Materials and Methods

This retrospective study was performed at the Erasmus MC Sophia Children’s Hospital, Rotterdam, Netherlands. The study did not require the approval of the local Human Research Ethics Committee because of the retrospective nature of the study and the anonymized laboratory data collection (personal and medical information of patients were not collected, except for age and sex). Salivary and serum cortisol values were provided by the Laboratory of Endocrinology with the support of the Informatics Department.

All salivary and serum cortisol values measured at the Laboratory of Endocrinology at Erasmus MC from 2015 to 2020 were obtained. The data were filtered according to the age, selecting patients aged 0–18 years. Only salivary and serum cortisol values measured during an ACTH stimulation test were included.

According to the internal protocol, HDT and LDT present different indications. The HDT is performed for differential diagnosis of primary adrenal insufficiency, suspicion of the adrenal enzymatic defect and differential diagnosis of hyperandrogenism (to distinguish between an adrenal or gonadal androgen production). The LDT test is indicated in patients suspected of secondary or tertiary adrenal insufficiency [16]. For the HDT, synthetic ACTH (Synacthen^®^) is administered i.v. at the standard dose of 250 µg in patients aged more than 2 years, while babies from 0 to 6 months receive 62.5 µg and infants aged from 6 months to 2 years receive 125 µg. Patients are not required to fast before the test and it can be performed in any moment of the day, however, preferably in the morning. If the patient is treated with steroids, the drug administration must be stopped in advance (12 h before the test for hydrocortisone and 3 days before for prednisone). During the test, the blood is collected in serum separator tubes before the administration of Synacthen^®^ (time 0, t0) and after 30 and 60 min (t30, t60). The LDT is performed with Synacthen^®^ i.v. at the dosage of 1 µg/kg in newborns and 1 µg/1.73 m² in older children. The desired dose is obtained after dilution of a vial of Synacthen^®^ 0.25 mg in 500 mL of NaCl 0.9%, leading to a concentration of 0.25 µg of Synacthen^®^ per ml of solution. The blood is drawn before the administration of Synacthen^®^ and after 10, 20, 30, 40 and 60 min for a total of 6 samples per test (t0, t10, t20, t30, t40, t60), according to the internal guidelines.

For both HDT and LDT, salivary samples are collected at the same time as blood withdrawal. The tubes used to collect saliva are called Salivette and they need to be filled with 300 µL of saliva without foam. The patients are asked to not drink, eat or brush teeth starting 30 min before the saliva collection. The adrenal function is considered sufficient if the peak of serum cortisol is above 420 nmol/L. No cut-off has been established yet for the salivary cortisol.

Salivary cortisol was measured by ultra-performance liquid chromatography coupled with mass spectroscopy (UPLC-MS/MS),Waters TQS, Waters, Etten-Leur, The Netherlands. Serum cortisol was measured using an automated immunoassay (Siemens Immulite 2000XPi, Siemens, Erlangen, Germany) which was calibrated to gas chromatography-isotope dilution mass spectrometry (GC-IDMS) using the ERM-DA451 IFCC Cortisol Reference Serum Panel (European Reference Materials according to the International Federation of Clinical Chemistry) or by UPLC-MS/MS (Waters TQS, Waters, Etten-Leur, The Netherlands), which was calibrated in the same way.

Statistical analysis and graphic elaboration were performed with Prism GraphPad software. Data distribution was tested through the Shapiro–Wilk test. The correlation between serum and salivary cortisol was determined according to the Pearson correlation coefficient. For the HDT, the correlation between serum and salivary cortisol was evaluated at t0, t 30, t60 and between peak values. For the LDT, the correlation between serum and salivary cortisol was investigated at t0 and at the peak.

We must specify that for 3 patients who underwent the HDT, the value of salivary cortisol at baseline was <1 nmol/L and the corresponding values of serum cortisol ranged from 97 to 121 nmol/L. In the case of the LDT, for 10 patients, the value of salivary cortisol at baseline was <1 nmol/L and the corresponding values of serum cortisol ranged from 28 to 122 nmol/L. Moreover, in the LDT, 2 patients presented basal serum cortisol <28 nmol/L and corresponding salivary cortisol 0.7 and 0.4 nmol/L. This discrepancy was related to the lower detection limit of the assay.

For the calculation of the correlation coefficient, when salivary cortisol was <1 nmol/L a value of 1 nmol/L was imputed; the correspondent serum cortisol was respected. The same was applied to serum cortisol <28 nmol/L, imputed as 28 nmol/L. To verify the validity of our choice, we calculated the correlation coefficient at baseline for the LDT pairing all salivary cortisol values <1 nmol/L (set at 1 nmol/L) with a serum cortisol value of 28 nmol/L. The correlation coefficients were comparable.

The distribution of salivary peak values in AI and AS patients was analyzed according to the interquartile distribution and plotted in a box and whisker diagram. The trend of serum and salivary cortisol during both the high- and low-dose ACTH was shown in a box and whisker diagram. Sensitivity and specificity of the salivary cortisol compared to the serum cortisol during the gold standard ACTH stimulation test were evaluated using as cut-off value for AI diagnosis a salivary cortisol < 15 nmol/L. This value value was derived from the interquartile distribution of salivary peak values of cortisol in AI and AS patients, and it was supported by the study of Cornes et al. who proposed the same value as cut-off [17].

## 3. Results

In the reference period 2015–2020, a total of 80 ACTH stimulation tests (characterized by simultaneous serum and salivary cortisol measurements) were obtained. The yearly distribution of the tests was: 19 ACTH stimulation tests in 2015, 12 ACTH stimulation tests in 2016, 6 ACTH stimulation tests in 2017, 21 ACTH stimulation tests in 2018, 10 ACTH stimulation tests in 2019, 12 ACTH stimulation tests in 2020. The tests were obtained from 75 different patients (5 patients underwent the test twice, in different years). The age ranged from 5 to 18 years. The population consisted of 36 males and 39 females. Main results are summarized in Table 1.

### 3.1. High-Dose ACTH Stimulation Test

The number of tests included was 24. All tests showed a sufficient serum cortisol peak. For 3 patients at t0, 1 patient at t30 and 3 patients at t60 samples of salivary and/or serum cortisol were not collected during the test or the collected material was insufficient.

**Figure 1:**
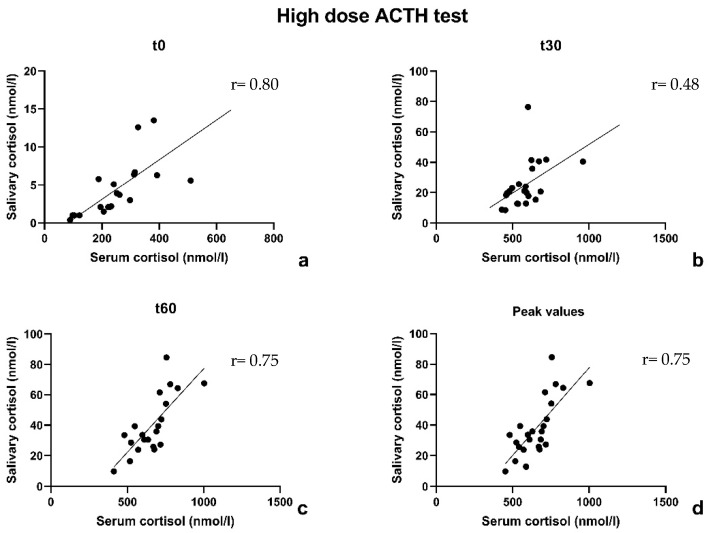
High-dose ACTH stimulation test: correlation between serum and salivary cortisol according to Pearson coefficient at t0 (**a**), at t30 (**b**), at t60 (**c**) and at the peak (**d**).

The correlation coefficient between serum and salivary cortisol was 0.80 at t0, 0.48 at t30 and 0.75 at t60. In 75% of cases, both serum and salivary cortisol peaks were reached 60 min after the Synacthen^®^ administration. In 3 cases (12.5%), the peak of serum and salivary cortisol occurred both 30 min after the Synacthen^®^ administration; in 2 cases (8.3%) the peak of serum cortisol was registered after 30 min while the salivary cortisol occurred after 60 min. The correlation coefficient between serum and salivary cortisol peaks was 0.75, which is in line with the coefficient calculated at t60. The data are shown in Figure 1.

### 3.2. Low-Dose ACTH Stimulation Test

The number of tests included was 56; in 41%, the peak of serum cortisol was indicative of insufficient adrenal function. For 7 patients, samples of salivary and/or serum cortisol at baseline were not collected during the test or the collected material was insufficient.

**Figure 2:**
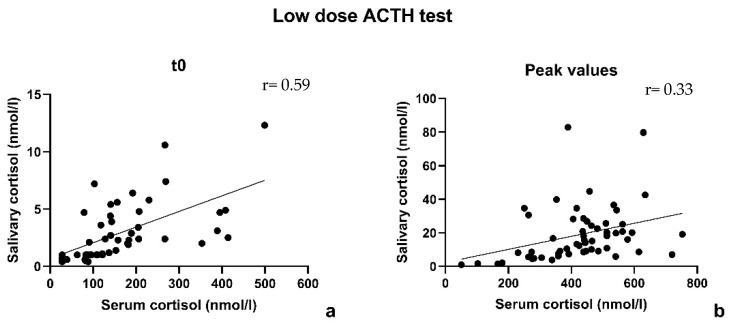
Low-dose ACTH stimulation test: correlation between serum and salivary cortisol according to Pearson coefficient at t0 (**a**) and at the peak (**b**).

The correlation coefficient between serum and salivary cortisol at t0 was 0.59. The correlation coefficient between serum and salivary cortisol at the peak was 0.33 (after excluding 3 pairs because of a not optimal match in sampling time, the coefficient improved only weakly, r = 0.41). The data are shown in Figure 2.

### 3.3. Comparison of Salivary Cortisol Peak Values in Adrenal Insufficient and Adrenal Sufficient Patients and Evaluation of Sensitivity and Specificity of the Derived Salivary Cortisol Cut-Off

Patients presenting a serum cortisol peak value > 420 nmol/L during the ACTH stimulation test were considered adrenal sufficient [8]. A serum cortisol peak value < 420 nmol/L allowed to make the diagnosis of AI.

**Figure 3:**
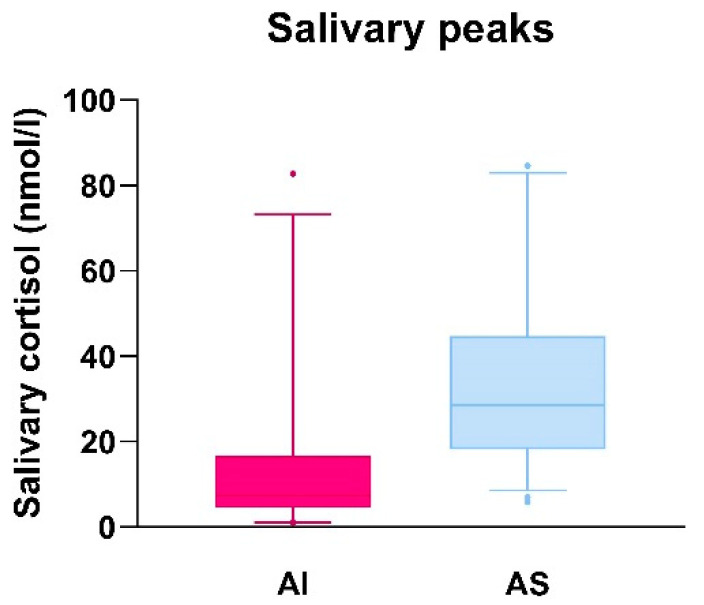
Salivary cortisol peak values in adrenal insufficient (AI) and adrenal sufficient (AS) patients plotted in a box and whisker diagram.

In Figure 3, the salivary cortisol peak values reached by AI patients (on the left) and AS patients (on the right) are plotted in a box and whisker diagram. The interquartile distribution is shown with a range from 5° to 95° percentile. For AI patients, the first quartile (Q1) corresponded to salivary cortisol of 4.65 nmol/L, the third quartile (Q3) to 15 nmol/L; the median was 7.4 nmol/L but the absolute range of values was wide (minimum value 1 nmol/L, maximum value 82.8 nmol/L). In AS patients, Q1 corresponded to a salivary cortisol value of 15.55 nmol/L, Q3 to 36.08 nmol/L; the median was 25.35 nmol/L. Also in this group, the range between the minimum and the maximum value was wide (5.9–84.6 nmol/L). The interquartile range was smaller for the AI patients (10.35) while wider for AS patients (25.53). In consideration of the aforementioned data, a peak value of salivary cortisol between 15 and 16 nmol/L (corresponding to AI Q3 and AS Q1) could be suggested as a cut-off point. Nevertheless, as shown in the diagram, due to the wide distribution of salivary cortisol peak values, this cut-off would underestimate adrenal function in some AS patients and overestimate it in some AI patients.

Sensitivity and specificity of salivary cortisol compared to serum cortisol were evaluated only for the LDT, because in the group of patients who underwent HDT, AI was not diagnosed. In the current study, the cut-off for salivary cortisol was set at <15 nmol/L, derived from the interquartile distribution. This cut-off was also suggested by the study of Cornes et al. [17].

For this cut-off, a sensitivity of 73.9% and a specificity of 69.6% were found. The area under the curve (AUC) was 0.78, C.I. 95% 0.64–0.93, *p*-value = 0. The positive predictive value (PPV) was 62.9% and the negative predictive value (NPV) was 79.3%.

### 3.4. Serum and Salivary Cortisol Time Response to ACTH Administration

**Figure 4:**
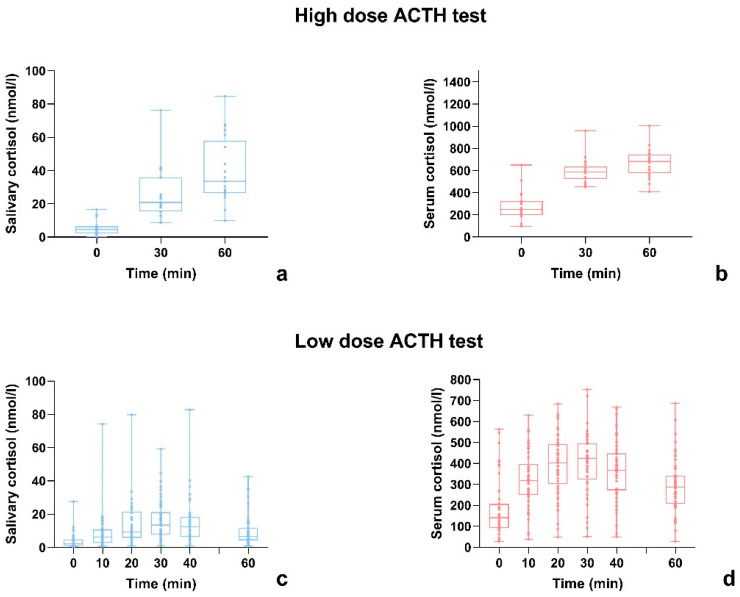
Serum and salivary cortisol time response to ACTH administration where (**a**,**b**) show the trend of salivary and serum cortisol during HDT, respectively and (**c**,**d**) show the trend of salivary and serum cortisol during LDT, respectively.

The trend of serum and salivary cortisol during both HDT and LDT is shown in Figure 4. If in the HDT both serum and salivary cortisol peaks tended to occur 60 min after the administration of Synachten^®^, in the LDT, the peaking time was less consistent: the cortisol peak was usually registered between 20 and 40 min after Synacthen^®^ administration, both for serum and salivary cortisol. At an individual level, serum and salivary cortisol did not reach the peak value exactly at the same moment; on the other hand, it was not possible to register a constant delay between serum and salivary peak.

## 4. Discussion

In the present study, the comparison of salivary cortisol with serum cortisol response to the ACTH test showed different results for the HDT and the LDT. Regarding the HDT, the peak of serum cortisol was mostly registered 60 min after Synacthen^®^ administration, in line with previous findings [12,18]. The HDT correlation coefficients between serum and salivary cortisol were strong, calculated at baseline (r = 0.80) and at peak (r = 0.75), respectively. All subjects tested resulted AS, with peak serum cortisol values ranging from 452 to 1004 nmol/L and salivary cortisol values ranging from 9.9 and 84.6 nmol/L. The LDT showed peak levels at t = 30 on average, in line with literature [19,20] and a moderate to weak correlation between serum and salivary cortisol at baseline (r = 0.59) and at the peak (r = 0.33), respectively; 41% of patients resulted AI (serum cortisol < 420 nmol/L), while the values of salivary cortisol in these AI patients varied in a wide range (1–82.8 nmol/L).

In literature, there is no agreement on the reliability of salivary cortisol during the LDT. If some authors already affirmed that in the LDT the sensitivity and specificity of the salivary cortisol are too low to represent an alternative to serum cortisol [21], more encouraging results have been shown in other cases [19,20].

Vaiani et al. used LDT to assess central AI in 145 children. The authors showed a positive correlation between stimulated serum cortisol and stimulated salivary cortisol, but not for the values at baseline. In consideration of considerable overlap of salivary cortisol values between AS and AI patients, they suggest that measuring only salivary during LDT is not useful to assess AI, but it could be helpful to differentiate an intermediate group of patients from a real insufficient one, avoiding unnecessary treatment [22].

In the current study, it was not possible to define a threshold for salivary cortisol to discriminate among AI and AS patients in the cohort that underwent HDT because all children were adrenal sufficient. However, in the cohort of patients examined with LDT we proposed a cut-off of 15 nmol/L to differentiate AI and AS patients according to the salivary cortisol. The sensitivity obtained was almost 74%, while the specificity was lower (69.6%). These values were not satisfactory, above all, if compared with data previously shown in literature in which the sensitivity and specificity reached 90–100% for similar cut-offs (13.2 and 13.8 nmol/L) [13,15].

In our cohort, 5 patients among the 23 AI presented a salivary cortisol peak higher than expected, for which we can only hypothesize that abnormal CBG levels or contamination of the salivary sample with blood played a role.

Regarding the cross-reactivity of immunoassays, a special consideration must be conducted on cortisone. Salivary cortisone derives from enzymatic conversion of salivary cortisol by 11β-hydroxysteroid dehydrogenase type 2 (11β-HSD-2) and from passive diffusion of free serum cortisone into the saliva. Salivary cortisone shows a stronger correlation with serum cortisol than salivary cortisol during an ACTH stimulation test at 60 min, while at baseline and early stages of the test, both salivary cortisol and salivary cortisone correlate poorly with serum cortisol [23]. Moreover, 11β-HSD-2 expression could vary among different individuals, and this could contribute to the absence of linear correlation between salivary and serum cortisol in a part of our cohort.

It is also important to highlight the possible interference in salivary cortisol determination depending on the system used to collect saliva. Kidd et al. demonstrated that in 16 healthy adult subjects, collection of saliva through cotton and polyester swabs led to an overestimation of salivary cortisol, while stimulation of salivary flux with citric acid did not appear to affect salivary cortisol levels, which were comparable to plain saliva (passive drooling in a container) [24]. Conversely, other authors demonstrated a stronger correlation between serum cortisol and salivary cortisol collected by swabs rather than plain saliva [25]. Also, high correlation was demonstrated among salivary cortisol levels measured after collecting saliva by passive drooling and by citric acid-treated swabs with plasma levels and among each other [26]. Finally, the process of sample collection, low volume saliva samples, the storage and the handling of samples can potentially influence the accuracy of the measurement of salivary cortisol [27].

The current study presents some points of strength such as the wide number of patients included, the focus on a pediatric population and the comparison between HDT and LDT. One of the factors that should be taken into account is the timing and frequency of sampling, as the LDT protocol differs greatly among the centers. A recent study performed by Gill et al. proposes to take cortisol samples at 0, 15, 30 and 60 min instead of only at 0 and 30 min as performed in most centers, showing that not performing 15 and 60 min samples would lead to 9.8% of misdiagnosis [28]. Our protocol, on the other hand, presents an even tighter timing of samples and, as shown in Figure 4, for both serum and salivary cortisol, the peak was usually registered between 20 and 40 min after ACTH administration. Special attention should be paid to the method used in cortisol determination. The main two are immunoassays (as in our case) and LC-MS/MS. Immunoassays are the most used due to their simple usage and high level of automation. Among the main limitations of immunoassays, there are the reproducibility (in our center, the variation coefficient is around 7%) and the significant antibody cross-reactivity with other analytes such as cortisone, which is present in the saliva in a concentration three to ten times higher than cortisol. On the other hand, mass spectrometry assays have a higher specificity, however, no single reference range has been validated [10,11].

Among the main limitations of the current study, we must include the retrospective design of the study and the choice to test with HDT patients suspected of CAH, while with LDT patients suspected of AI (primary or central). We can infer that the group of children that underwent HDT had a low a priori chance of AI. This could explain, at least partially, the differences found in the correlation between serum and salivary cortisol during HDT and LDT. Moreover, for some patients, a high discrepancy between serum and salivary cortisol response to the ACTH stimulation test was registered and this could be explained by low levels of CBG or underlying diseases.

## 5. Conclusions

In conclusion, at the moment, there is a lack of univocal data to support the use of salivary cortisol as an alternative to serum cortisol during an ACTH stimulation test. Our data do not support salivary cortisol as a valid alternative to serum cortisol during the LDT. With regard to the HDT, data about the correlation between salivary and serum cortisol are more encouraging but further studies are needed. Ideally, patients with a high a priori chance of AI should be tested with HDT to define a cut-off value for salivary cortisol also. However, the differences in protocols to collect saliva, laboratory assays and the absence of uniform cut-off values are still main points of discussion and therefore, the possibility that every center should define its own cut-off value is consistent.

## Figures and Tables

**Table 1:** Main results are summarized in the table below.

	HDT (*n* = 24)	LDT (*n* = 56)
Adrenal insufficient tests	0	23
Peak time	60 min	20–40 min
R coefficient at t0	0.80	0.59
R coefficient at peak	0.75	0.33
Sensitivity *	NA	73.9%
Specificity *	NA	69.6%
Positive predictive value *	NA	62.9%
Negative predictive value *	NA	79.3%

* Calculated for a salivary cortisol cut-off of 15 nmol/L. NA: not applicable.

## Data Availability

All data generated or analyzed during this study are included in this published article.

## References

[1] Husebye E.S., Pearce S.H., Krone N.P., Kämpe O. (2021). Adrenal insufficiency. Lancet.

[2] Chabre O., Goichot B., Zenaty D., Bertherat J. (2017). Group 1. Epidemiology of primary and secondary adrenal insufficiency: Prevalence and incidence, acute adrenal insufficiency, long-term morbidity and mortality. Ann. Endocrinol..

[3] Betterle C., Presotto F., Furmaniak J. (2019). Epidemiology, pathogenesis, and diagnosis of Addison’s disease in adults. J. Endocrinol. Investig..

[4] Wijaya M., Huamei M., Jun Z., Du M., Li Y., Chen Q., Chen H., Song G. (2019). Etiology of primary adrenal insufficiency in children: A 29-year single-center experience. J. Pediatr. Endocrinol. Metab..

[5] Eyal O., Levin Y., Oren A., Zung A., Rachmiel M., Landau Z., Schachter-Davidov A., Segev-Becker A., Weintrob N. (2019). Adrenal crises in children with adrenal insufficiency: Epidemiology and risk factors. Eur. J. Pediatr..

[6] Chung S., Son G.H., Kim K. (2011). Circadian rhythm of adrenal glucocorticoid: Its regulation and clinical implications. Biochim. Biophys. Acta—Mol. Basis Dis..

[7] Bornstein S.R., Allolio B., Arlt W., Barthel A., Don-Wauchope A., Hammer G.D., Husebye E.S., Merke D.P., Murad M.H., Stratakis C.A. (2016). Diagnosis and treatment of primary adrenal insufficiency: An endocrine society clinical practice guideline. J. Clin. Endocrinol. Metab..

[8] El-Farhan N., Pickett A., Ducroq D., Bailey C., Mitchem K., Morgan N., Armston A., Jones L., Evans C., Rees D.A. (2013). Method-specific serum cortisol responses to the adrenocorticotrophin test: Comparison of gas chromatography-mass spectrometry and five automated immunoassays. Clin. Endocrinol..

[9] Ospina N.S., Nofal AAl Bancos I., Javed A., Benkhadra K., Kapoor E., Lteif A.N., Natt N., Murad M.H. (2016). ACTH stimulation tests for the diagnosis of adrenal insufficiency: Systematic review and meta-analysis. J. Clin. Endocrinol. Metab..

[10] El-Farhan N., Rees D.A., Evans C. (2017). Measuring cortisol in serum, urine and saliva—Are our assays good enough?. Ann. Clin. Biochem..

[11] Bastin P., Maiter D., Gruson D. (2018). Salivary cortisol testing: Preanalytic and analytic aspects. Ann. Biol. Clin..

[12] Kosák M., Hána V., Hill M., Šimůnková K., Lacinová Z., Kršek M., Marek J. (2014). Serum cortisol seems to be a more appropriate marker for adrenocortical reserve evaluation in ACTH test in comparison to salivary cortisol. Physiol. Res..

[13] Kim Y.J., Kim J.H., Hong A.R., Park K.S., Kim S.W., Shin C.S., Kim S.Y. (2020). Stimulated Salivary Cortisol as a Noninvasive Diagnostic Tool for Adrenal Insufficiency. Endocrinol. Metab..

[14] Langelaan M.L.P., Kisters J.M.H., Oosterwerff M.M., Boer A.K. (2018). Salivary cortisol in the diagnosis of adrenal insufficiency: Cost efficient and patient friendly. Endocr. Connect..

[15] Chao C.S., Shi R.Z., Kumar R.B., Aye T. (2020). Salivary cortisol levels by tandem mass spectrometry during high dose ACTH stimulation test for adrenal insufficiency in children. Endocrine.

[16] Van der Kamp H.J., Kamphuis S.S.M., Merkus P.J.F.M., Schroor E.J. (2019). Richtlijn Afbouwen Glucocorticoïden bij Kinderen.

[17] Cornes M.P., Ashby H.L., Khalid Y., Buch H.N., Ford C., Gama R. (2015). Salivary cortisol and cortisone responses to tetracosactrin (synacthen). Ann. Clin. Biochem..

[18] Deutschbein T., Unger N., Mann K., Petersenn S. (2009). Diagnosis of secondary adrenal insufficiency in patients with hypothalamic-pituitary disease: Comparison between serum and salivary cortisol during the high-dose short synacthen test. Eur. J. Endocrinol..

[19] Šimůnková K., Hampl R., Hill M., Doucha J., Stárka L., Vondra K. (2007). Salivary cortisol in low dose (1 μg) ACTH test in healthy women: Comparison with serum cortisol. Physiol. Res..

[20] Marcus-Perlman Y., Tordjman K., Greenman Y., Limor R., Shenkerman G., Osher E., Stern N. (2006). Low-dose ACTH (1 μg) salivary test: A potential alternative to the classical blood test. Clin. Endocrinol..

[21] Schindhelm R.K., Van De Leur J.J.C.M., Rondeel J.M.M. (2010). Salivary cortisol as an alternative for serum cortisol in the low-dose adrenocorticotropic hormone stimulation test?. J. Endocrinol. Investig..

[22] Vaiani E., Lazzati J.M., Ramirez P., Costanzo M., Gil S., Dratler G., Zaidman V., Chaler E., Belgorosky A. (2019). The Low-Dose ACTH Test: Usefulness of Combined Analysis of Serum and Salivary Maximum Cortisol Response in Pediatrics. J. Clin. Endocrinol. Metab..

[23] Elder C.J., Harrison R.F., Cross A.S., Vilela R., Keevil B.G., Wright N.P., Ross R.J. (2018). Use of salivary cortisol and cortisone in the high- and low-dose synacthen test. Clin. Endocrinol..

[24] Kidd S., Midgley P., Lone N., Michael Wallace A., Nicol M., Smith J., McIntosh N. (2009). A re-investigation of saliva collection procedures that highlights the risk of potential positive interference in cortisol immunoassay. Steroids.

[25] Poll E.M., Kreitschmann-Andermahr I., Langejuergen Y., Stanzel S., Gilsbach J.M., Gressner A., Yagmur E. (2007). Saliva collection method affects predictability of serum cortisol. Clin. Chim. Acta.

[26] Gallagher P., Leitch M.M., Massey A.E., McAllister-Williams R.H., Young A.H. (2006). Assessing cortisol and dehydroepiandrosterone (DHEA) in saliva: Effects of collection method. J. Psychopharmacol..

[27] Harmon A.G., Hibel L.C., Rumyantseva O., Granger D.A. (2007). Measuring salivary cortisol in studies of child development: Watch out—What goes in may not come out of saliva collection devices. Dev. Psychobiol..

[28] Gill H., Barrowman N., Webster R., Ahmet A. (2019). Evaluating the Low-Dose ACTH Stimulation Test in Children: Ideal Times for Cortisol Measurement. J. Clin. Endocrinol. Metab..

